# Community PrEP delivery for adolescent girls and young women: insights from the DREAMS PrEP Choice Study in Johannesburg, South Africa

**DOI:** 10.3389/frph.2025.1474067

**Published:** 2025-04-04

**Authors:** Nicolette Prea Naidoo, Nqaba Mthimkulu, Ngcwalisa Jama, Glory Chidumwa, Brison Chabalala, Tafadza Mhakakora, Lauren Parmley, Saiqa Mullick

**Affiliations:** ^1^ Wits RHI, University of Witwatersrand, Johannesburg, South Africa; ^2^ United States Agency for International Development (USAID), Pretoria, South Africa

**Keywords:** HIV prevention, AGYW, PrEP, South Africa, community-based

## Abstract

**Introduction:**

Long-acting pre-exposure prophylaxis (PrEP) methods have the potential to significantly reduce HIV acquisition and increase the uptake and effective use of PrEP among adolescent girls and young women (AGYW). We describe the implementation approach for delivering the PrEP ring and outline provider perspectives related to community-based service delivery.

**Methods:**

The DREAMS PrEP Choice Study, a mixed-methods implementation science study delivering PrEP choice to women 18 years and older, was conducted in Johannesburg, South Africa. We extracted quantitative enrollment data from the routine patient management system (REDCap) and collected qualitative data from four focus group discussions with providers. Quantitative data are presented descriptively whilst qualitative data were analyzed using content and thematic analyses.

**Results:**

Between October 2023 and April 2024, 657 participants were enrolled and offered PrEP choice. Most (72.1%, *n* = 474) participants were between 18 and 24 years old and accessed services at education and training institutions (52.1%, *n* = 342). Furthermore, 68.8% (*n* = 451) chose oral PrEP at enrollment, 26.6% (*n* = 175) chose the PrEP ring, and 3.2% (*n* = 20) selected no method. Most participants accessed services through a mobile truck (*n* = 365, 55.6%). There was a strong preference for nurse insertion of the ring on site (*n* = 103, 58.9%). Site setup, privacy, and access to running water, in alignment with national PrEP ring guidelines, were reported as barriers to implementation.

**Conclusion:**

As one of the first studies to implement community-based services for PrEP choice, the early results indicate the feasibility of delivering this service with moderate uptake of PrEP by AGYW. Understanding the service delivery conditions required to offer informed choice is important for supporting national scale-up.

## 1 Introduction

The Joint United Nations Program on HIV/AIDS reports that there are an estimated 29.8 million people living with HIV globally, with South Africa bearing the greatest burden (1). Despite the expansion of HIV care and treatment, HIV incidence remains high in eastern and southern African settings, particularly among adolescent girls and young women (AGYW) aged 15–24 years who account for an estimated 29% of all new HIV infections in the region. In 2024, the South African national HIV incidence was 0.39% among youth and young adults aged 15–24 years. Although HIV prevalence has decreased since 2017, compared to men, HIV prevalence was approximately twofold higher in girls and women aged 15–19 (5.7% vs. 3.1%) and 20–24 years (8.0% vs. 4.0%), and threefold higher in women aged 25–29 years (19.5% vs. 6.3%) (2).

Oral tenofovir disoproxil fumarate (TDF)-based pre-exposure prophylaxis (PrEP), a daily oral pill, is highly protective against HIV acquisition (3). In 2015, the WHO recommended that daily oral PrEP be provided to people with a substantial risk of acquiring HIV (4). AGYW were identified as having substantial risk of HIV acquisition and prioritized as recipients of PrEP. In 2016, the South African National Department of Health (NDOH) began offering oral PrEP to female sex workers (FSW) and men who have sex with men (MSM), and in 2018, to AGYW. Since then, significant strides have been made to increase access to oral PrEP, with the NDOH reporting 1.7 million people to have been initiated on oral PrEP by the end of October 2024 across 96% of public sector health facilities, the majority (52%) of which are girls and women aged 15 to 24 years (5).

However, despite the increased scale-up, gaps still remain in reaching those most in need, particularly in high-incidence areas, in achieving optimal facility PrEP coverage, in supporting continuation and effective use, and in increasing social mobilization and demand creation for both PrEP and post-exposure prophylaxis (5). Studies have reported difficulty with daily oral pill taking and low continuation among AGYW (6), and structural barriers make access to services difficult (7). Therefore, to ensure increased reach and uptake, PrEP will need to be delivered beyond public facilities and in longer-acting formulations to make it easier to adhere to. The WHO recommends simplified and differentiated service delivery for HIV testing and antiretroviral therapy (ART), allowing for more person and community-centered care that is responsive to client needs and thereby reducing opportunity costs and removing barriers to access (8), particularly for adolescents who show little to no health-seeking behavior due to interpersonal and structural factors (9). Studies highlight that community-based oral PrEP delivery is feasible and acceptable to AGYW in various settings (10, 11) but the diversification of locations and delivery of long-acting PrEP methods is required to meet AGYW prevention needs. As countries explore models of service delivery, evidence is required to inform program decisions on approaches for the delivery of new methods and implementation of PrEP choice, and continuation of PrEP, particularly among AGYW (9). This paper provides critical insight into the practical considerations for offering PrEP choice across varied community settings and approaches.

Noting the essential role of PrEP in reducing new infections, maintaining adequate adherence and persistence over time is one of the greatest challenges of PrEP implementation. The dapivirine vaginal ring (PrEP ring) is a flexible silicone ring inserted into the vagina that slowly releases the antiretroviral dapivirine over 28 days of continuous use, after which it is replaced with a new ring. The PrEP ring prevents HIV acquisition from receptive vaginal sex only. This method is user-initiated and may present an acceptable option for clients who cannot or do not wish to take oral PrEP. Evaluated in clinical trials, the PrEP ring has been found to provide up to 50% efficacy in protecting against HIV acquisition. The clinical trials found higher HIV prevention efficacy for the PrEP ring in women who had greater drug release from their rings, reflecting more consistent use (12). New long-acting methods such as the PrEP ring may offer adherence benefits as global data indicate a decline in PrEP use over time, particularly amongst AGYW (13). Additionally, the expansion of PrEP product choice may increase overall uptake and utilization of PrEP, as was seen with the uptake of contraception with the expansion of contraceptive method choice (14). In more recent findings, and specific to HIV prevention, the SEARCH trial conducted in rural Uganda and Kenya demonstrated that HIV prevention choice increases coverage and protection at the population level (15).

Following the WHO's recommendation that a PrEP ring be offered as an additional HIV prevention option for women in January 2021, South Africa approved PrEP rings for use in March 2022 and rolled out clinical and implementation guidelines and training by 2023. According to the South African guidelines, a PrEP ring is permitted for use only among women 18 years and older, excluding those who are pregnant, breastfeeding, or younger than 18 years of age (16).

Historically, the introduction of evidence-based technologies into public health programs has been fraught with delays that result in missed opportunities for impact (17). These delays have in part been due to a lack of preparatory evidence critical for introduction into programs. For long-acting HIV prevention methods to be introduced without delay, key questions must be addressed, including how to scale products efficiently and effectively for people at risk of HIV infection. Building upon lessons learned from oral PrEP scale-up, operational research and implementation science are crucial to prepare the field for the delivery of new longer-acting PrEP products, which offer a more discrete option for users.

Since October 2019, Wits Reproductive Health and HIV Institute (RHI), through a US President's Emergency Plan for AIDS Relief award under the United States Agency for International Development’s (USAID) Determined, Resilient, Empowered, AIDS-free, Mentored, and Safe (DREAMS) program, has implemented and scaled mobile PrEP services for at-risk AGYW across fourteen districts in South Africa. PrEP services are offered as part of a broader package of sexual and reproductive health (SRH) services, in accordance with the NDOH's Guidelines for the Provision of Pre-exposure Prophylaxis to Persons at Substantial Risk of HIV Infection (18). Between November 2019 and 30 June 2024, 250,813 beneficiaries received PrEP through the award, of which 88% were AGYW. This equates to 18% of the national cumulative PrEP achievement (5). Community-based implementation incorporates targeted demand-creation activities to support normalization and buy-in of PrEP as an effective prevention tool. This includes the delivery of health promotion talks and the use of social media, community dialogues, and community radio to raise awareness of and facilitate linkage to PrEP. Services are located near targeted institutions of higher learning and at community sites with a high volume of AGYW and are provided either through a mobile clinic or pop-up gazebo. Activities are implemented to find and engage vulnerable adolescents and AGYW for HIV testing and SRH and PrEP services through community entry points.

Leveraging the existing Wits RHI community-based PrEP program, we nested an implementation science study to generate real-world evidence on the provision of PrEP choice (oral PrEP and PrEP ring). These findings will inform product introduction and implementation strategies for national scale-up, whilst also contributing to the growing evidence base on the impact of differentiated service delivery models for PrEP delivery (19). This paper describes the approach to delivering SRH and PrEP choice (oral PrEP and PrEP ring) to AGYW through a community-based implementation science study (DREAMS PrEP Choice) in Johannesburg, South Africa.

## 2 Methods

### 2.1 Research setting and study participants

The implementation research study (referred to as the “DREAMS PrEP Choice Study” in communities) is a prospective observational cohort study comprised of mixed methods (quantitative structured interviews, in-depth interviews, focus group discussions, and routine service delivery data). This paper only reports on the findings from four focus group discussions and routine service delivery data. The study was implemented in 38 study sites spread across seven sub-districts located in Johannesburg Health District, Gauteng Province, South Africa, with active enrollment between October 2023 and April 2024, and follow-up through to September of the same year. The sub-districts varied by setting (urban and peri-urban), socioeconomic status (indicating a vast contrast between poverty and wealth), and population density. Sites were selected based on existing relationships for program delivery and included sites frequented by AGYW (second chance matric centers, higher learning institutions, and community skills and youth centers).

All sexually active women 18 years and older accessing PrEP and/or SRH services at community-based outreach sites in Johannesburg were invited to participate in the study provided they met the eligibility criteria: female sex assigned at birth, receiving SRH and or PrEP services at the community-based site, HIV negative status confirmed through a rapid point of care antibody/antigen test, 18 years or older, not pregnant at enrollment, willing and able to initiate PrEP ring or oral PrEP and participate in required study procedures, and willing and able to provide written informed consent. Utilizing a consecutive sampling approach, interested participants were invited by the fieldworker to screen for study eligibility. Only those meeting the study eligibility criteria were enrolled. Clinical and non-clinical providers involved in the provision of SRH and PrEP services were purposively selected to participate in the qualitative aspect of the study.

### 2.2 Implementation/intervention approach

**Figure 1:**
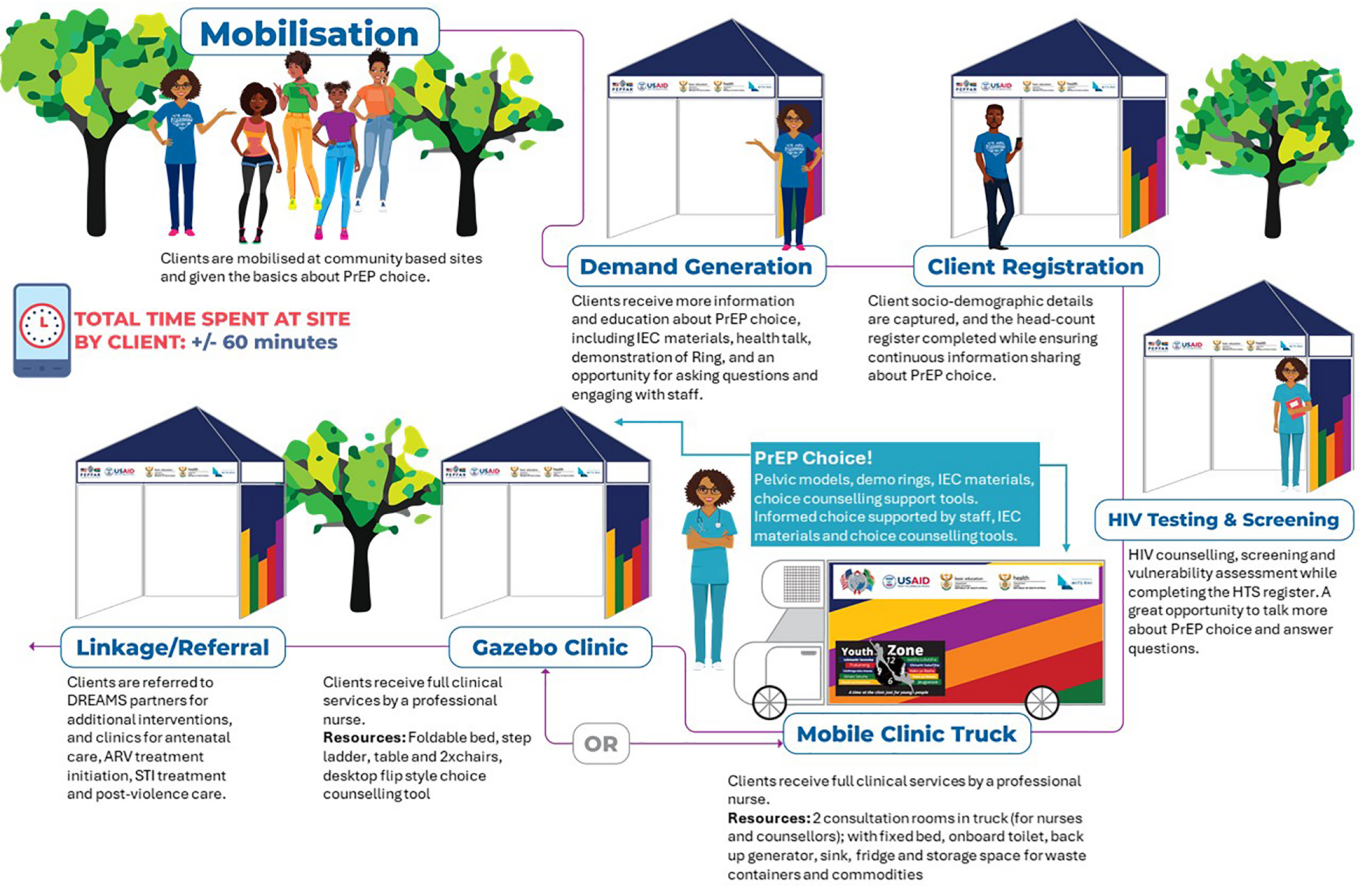
Visual schematic of the implementation delivery in community settings.

**Figure 2:**
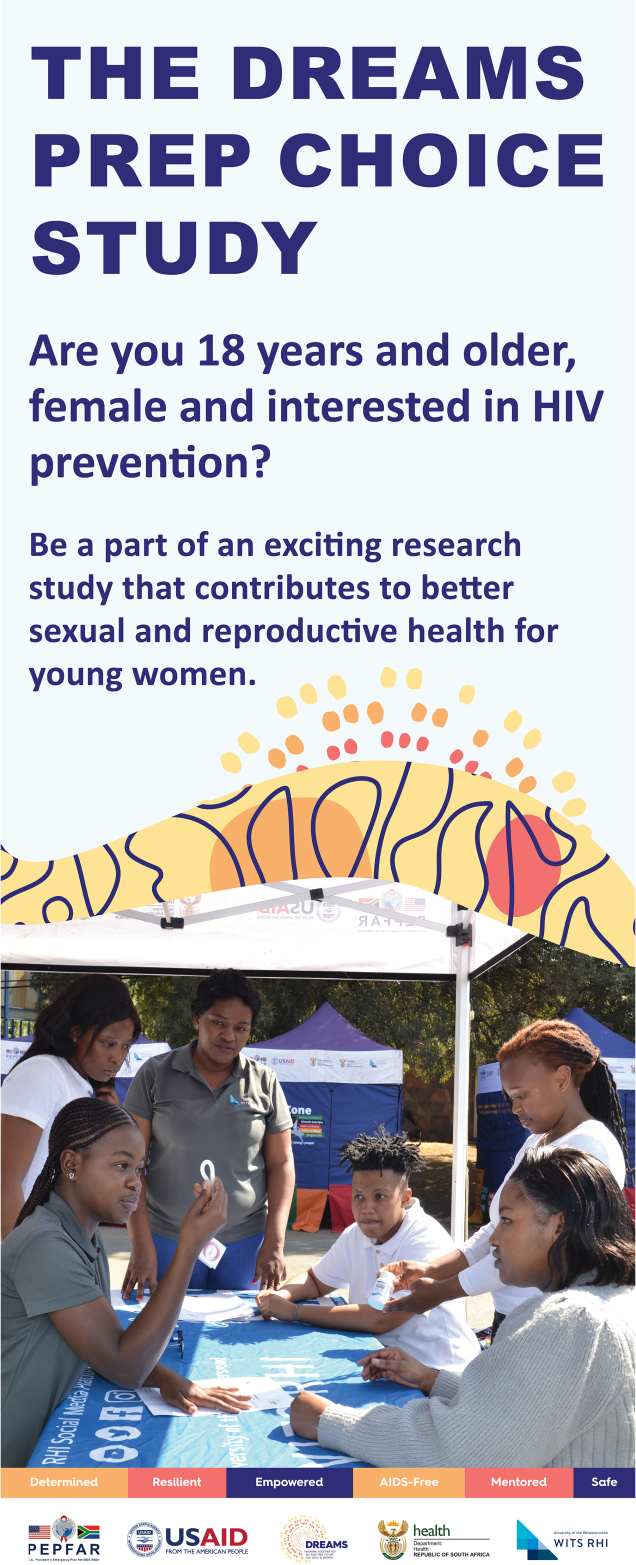
Study recruitment banner in English.

**Figure 3:**
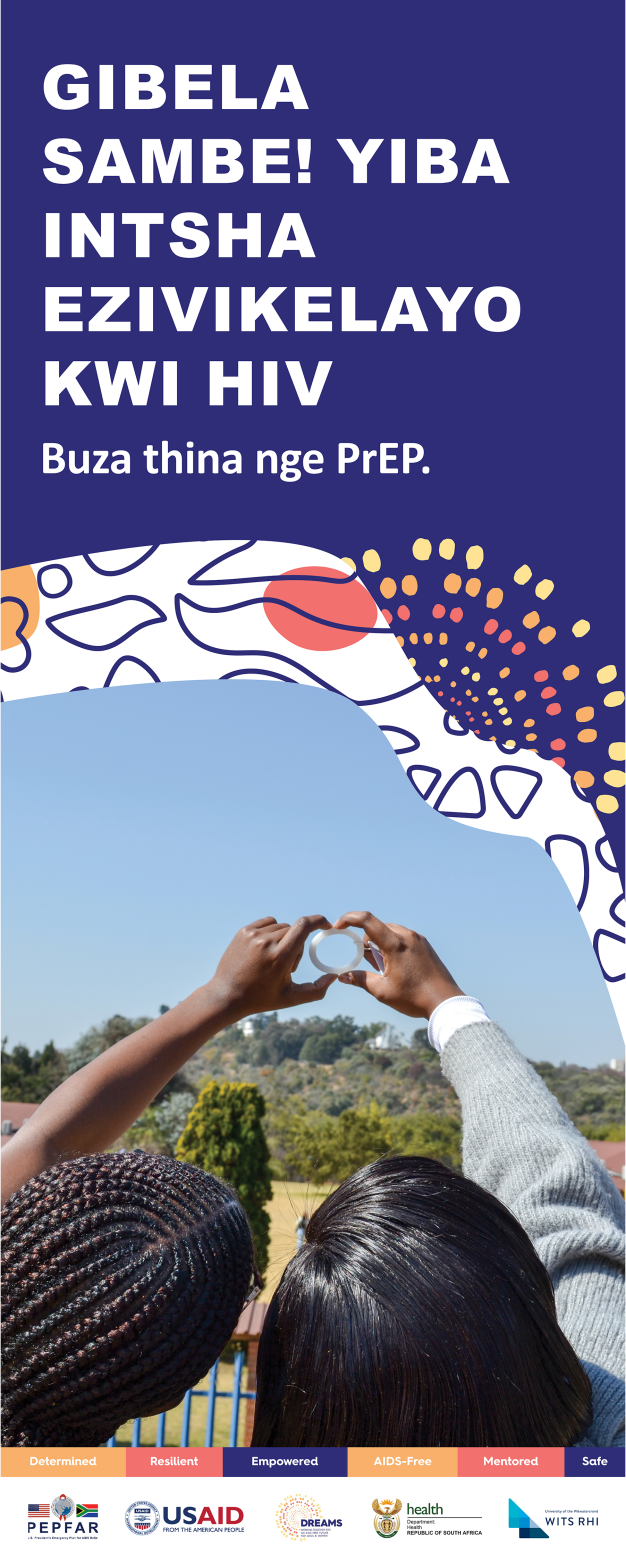
Study recruitment and PrEP awareness banner in Zulu language.

**Figure 4:**
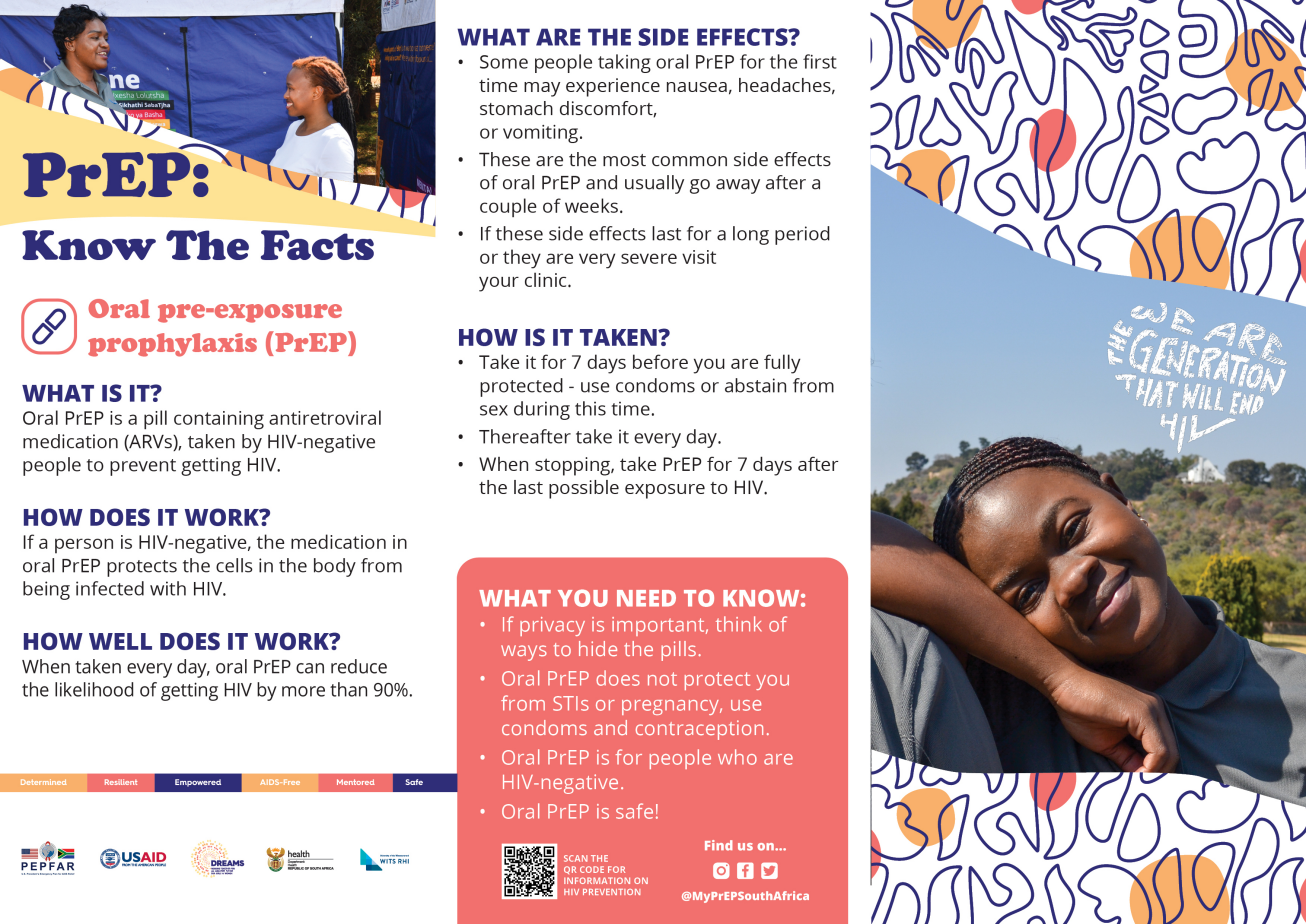
Oral PrEP factsheet shared with participants.

**Figure 5:**
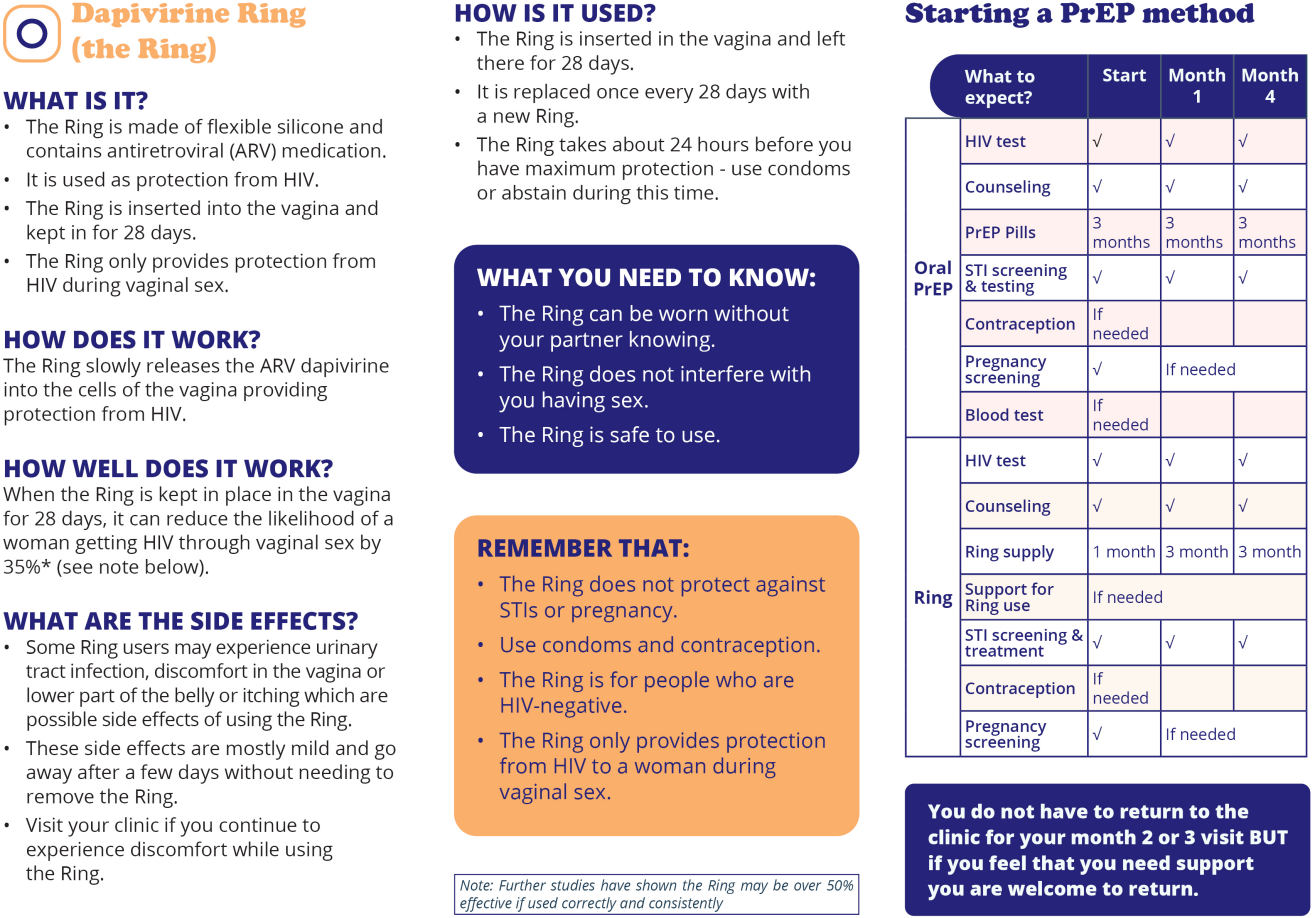
Dapivirine ring factsheet for participants.

The service delivery model included: (a) two mobile trucks with two consultation rooms each, (b) 22 pop-up gazebos to accommodate 36 providers (17 clinical and 19 non-clinical staff) to deliver clinical and non-clinical services. This model allowed for services to be delivered where beneficiaries are located, specifically community safe spaces (youth and skills centers and community-based organizations), colleges, universities, and student accommodation. Service delivery was informed by a weekly and 4-monthly scheduling roster which outlined the sites and frequency of visits (based on initial and follow-up visits). Under the DREAMS PrEP Choice Study, the PrEP ring was offered alongside oral PrEP service delivery. Trained healthcare providers offered PrEP choice (oral PrEP or PrEP ring) in line with the national guidelines and product eligibility along with SRH [pregnancy testing, sexually transmitted infection (STI) screening, and contraception and condom provision] services. Providers were trained on the NDOH guidelines for the implementation of the PrEP ring in South Africa, followed by weeks of pragmatic training, specifically practicing ring insertion and removal, choice counseling role-playing, and listening to end-user testimonials. The model of implementation is outlined in Figure 1. A core intervention package of HIV prevention including PrEP and SRH services was delivered through a roving mobile team comprising nurses, counselors, demand-creation officers/mobilizers, linkage officers/case navigators, and data capturers, all of whom were provided oversight by a clinical mentor. Demand generation and study recruitment banners, posters, and flyers were developed to mobilize participants and screen for eligibility (Figures 2–5).

As per Figure 1, AGYW presenting at community-based outreach sites were invited to participate in the study. Upon arrival at a site, the client was approached (either as a group or one-on-one) by a demand creation officer or mobilizer and provided with information on HIV testing, SRH, and PrEP (oral PrEP and PrEP ring). Where available, the clients were also shown a demonstration of the PrEP ring so the clients could feel and touch the product. The client was subsequently registered by the data-capturer. Following service registration, the client proceeded to the HIV counselor where a rapid HIV test was conducted, including pre and post-test counseling, along with an HIV risk and vulnerability assessment. This counselor also provided information on the two PrEP methods to assess the client’s interest and need for PrEP. Where the client showed interest, a fieldworker conducted study eligibility screening, including a pregnancy test and informed consent, thereafter linking the client to the professional nurse for SRH and PrEP services. PrEP was offered in accordance with national guidelines and includes blood/laboratory-based testing where indicated (16, 18). Specifically, oral PrEP was provided with baseline blood testing for hepatitis B and urine testing for kidney function, whereas the PrEP ring does not require blood tests. Screening for pregnancy does occur as the PrEP Ring is not recommended for use in pregnant and breastfeeding populations. Screening for STIs prior to PrEP initiation is recommended as severe ulcerations, pain, or discharge may delay PrEP Ring initiation until the symptoms have resolved (16). Follow-up visits for both oral PrEP and PrEP ring were scheduled 1 month after initiation and then quarterly thereafter, with a wellness check-in telephone call 7 days post-initiation.

**Figure 6:**
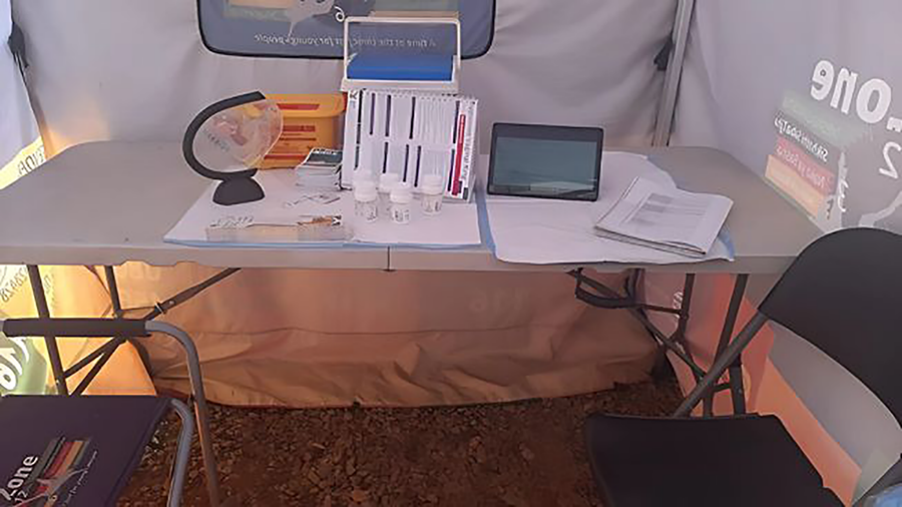
Nurse consultation room set up in a gazebo model for the delivery of oral PrEP and PrEP Ring in Communities.

Ring provision and choice counseling, conducted by the healthcare provider, were facilitated by the use of standardized national job aids that focused on the PrEP Ring initiation algorithm, follow-up visit schedules, readiness assessment, pregnancy testing and screening, and guidance on ring insertion and removal including understanding the pelvic area in relation to ring provision. The remaining job aids focused on informed decision-making and choice including a client information sheet introducing the ring to the participant (this was utilized in a participant-facing information pocket booklet) and key counseling and education messages (16). Providers approached counseling by trying to understand from participants which factors were important in their decision-making process when choosing an HIV prevention method. This included discussing with the participant their risk; partner disclosure and preference; frequency of sex (regular, unpredictable, or specific times); the effectiveness of the method (taken as prescribed: daily for oral pill and, for the ring, inserted and kept in place for a month until next clinic visit); personal commitment and preference for mode of use (pill or ring); side effect profile; protection against STIs, HIV, and pregnancy; clinical eligibility for the product; and personal preference. In addition to the above job aids, implementation staff (demand-creation officers conducting health talks, the HIV testing services (HTS) counselor also screening for PrEP, and the nurse provider) had a demonstration ring that could be used as an aid to increase awareness of the product. Additionally, nurse providers had a pelvic model that could be used to demonstrate the insertion and removal of the ring during the consultation. Participants also had the opportunity to feel the ring and practice inserting/removing it from the pelvic model as a practical exercise. It should be noted that oral PrEP was not demonstrated alongside the PrEP Ring but an image of the pill was shown on a job aid.

**Figure 7:**
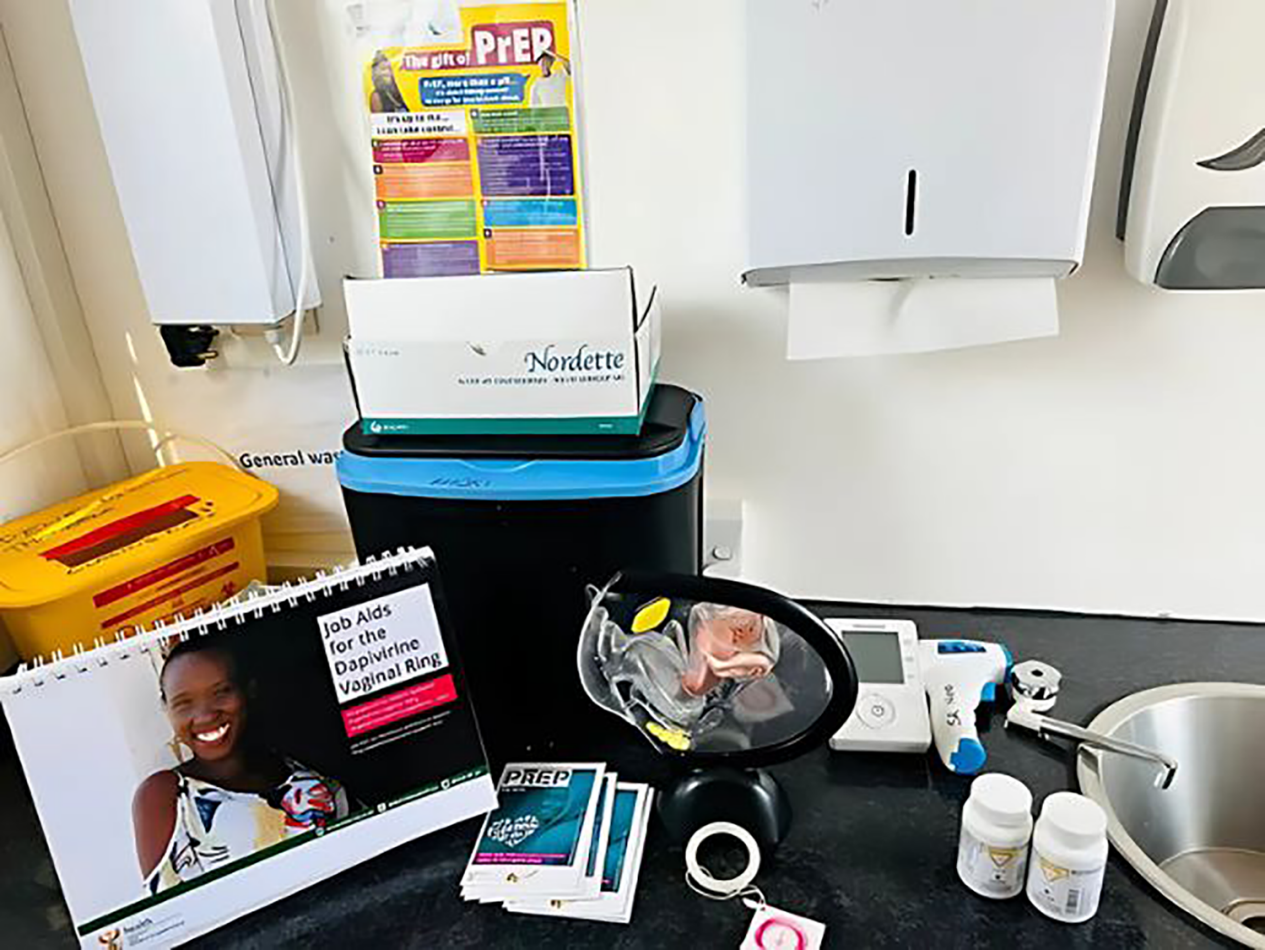
Mobile truck set up for the delivery of oral PrEP and PrEP ring.

**Figure 8:**
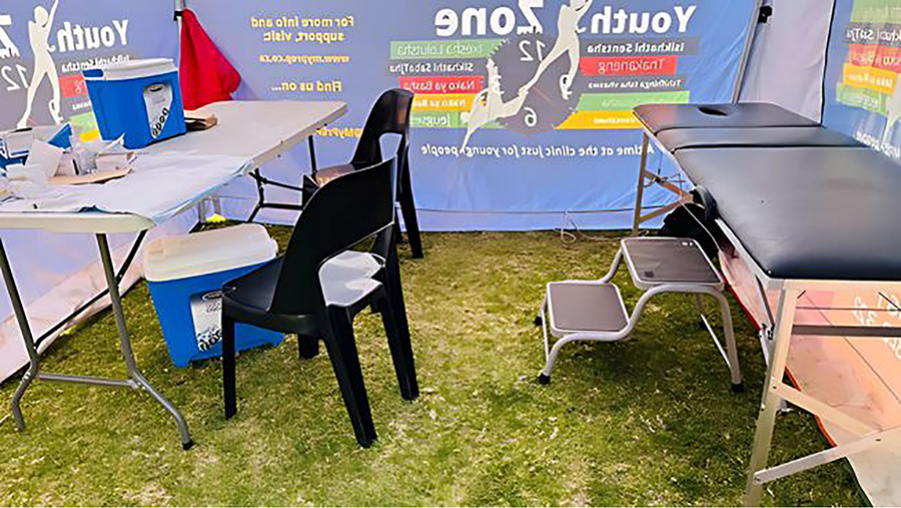
Nurse consultation room set up in a gazebo model for the delivery of oral PrEP and PrEP Ring in Communities.

Adapting the WHO framework for differentiated PrEP service delivery (8), we summarized the approach to implementation of the PrEP ring and oral PrEP with reference to the four building blocks (where, who, when, and what) with an update to the “where” to include “how”. Table 1 provides details regarding each building block. Additionally, site setup is shown in Figures 6–8.

**Table 1:** Description of implementation approach utilizing an adapted version of the WHO differentiated service delivery building blocks.

Building block	Demand creation (awareness and knowledge)	Client service registration	PrEP eligibility assessment	PrEP initiation, initial follow-up (0–3 months), re-initiation		PrEP continuation (3+ months)	
				Initiation/re-initiation	PrEP follow-up (0–3 months) as per PrEP method dosing schedule	PrEP refill	Follow-up
Where and how							
Services are delivered through either a mobile clinic or pop-up gazebos in a community setting and specifically designated safe spaces within communities (site types: skills and youth learning centers, higher learning institutions, technical and vocational colleges and universities, and student accommodation).	Clients are mobilized from within the respective community setting and the surrounding areas and gathered for health talks and demand generation activities (conducted at station 1 as per the implementation model outlined in Figure 1). This activity could be done under gazebos or by utilizing existing spaces such as outside lawns, community halls, classrooms, or amphitheaters.	Client sociodemographic data and data sharing consent to facilitate linkage to additional services are collected. Data collection is either paper-based or electronic tablet-based. This activity is conducted at station 2 as per the implementation model described in Figure 1). This is conducted in a gazebo.	Conducted in a mobile truck consultation room or pop-up gazebo. The placement of this station (station 3 as per the implementation model described in Figure 1) can be alongside other stations within the designated space.	Conducted in a mobile truck consulting room (includes bed) where feasible OR pop-up gazebo (foldable bed included). The placement of gazebos is important (identify appropriate space and layout to ensure confidentiality). Considered station 4 in the implementation model and can be multiple stations of the same kind depending on client volumes.	Conducted in a mobile truck consulting room (includes bed) OR pop-up gazebo (account for appropriate space and layout to ensure confidentiality). Same site as where initiation was done with the option for the client to be referred to a nearby site if unable to return (clients may initiate at a different site from where they live or frequent on a daily basis).	Same site as initiation (where feasible) or nearby site. Participant seen in either gazebo or mobile truck consulting room.	
Who?							
*Service provider*	Demand-creation officer, demand-creation mobiliser, community-based HIV prevention ambassador.	Data capturer (Opportunity to task shift this responsibility if staff numbers are reduced. For example, a case navigator would be able to perform this duty provided they have the minimum qualification.)	An HIV testing services (HTS) counselor and/or a professional nurse (pending volume of clients on site) able to perform HTS.	Professional nurse with a diploma in Nurse Initiation and Management of ART (NIMART) and dispensing license.			
When?							
*Service frequency (daily, weekly, monthly, every 3 months)*	Same day as service delivery AND a week before arrival at implementation site.	Same day and prior to PrEP assessment.	After same-day HIV testing; when other services are offered (contraception and STI screening).	Same-day PrEP initiation after HIV negative test result of 1-month prescription	1 month post-initiation in-person (same for oral PrEP and PrEP ring).	Every 3 months (same for oral PrEP and PrEP ring).	
What?							
Service package (demand creation, HIV testing, clinical monitoring, PrEP dispensing and comprehensive services)	Onsite face-to-face: as and when clients are mobilized for service delivery. Clients are provided with information on STIs, contraception, HTS, and PrEP (oral PrEP and PrEP ring). Demo ring available as a tool to increase awareness among beneficiaries; banners, pocket books, and flyers including QR codes to increase awareness of the study and PrEP services, offsite online engagement: targeted Facebook posts (mobile truck schedule), email, and WhatsApp communication with stakeholders/clients a week before service delivery.	Tablet or paper-based completion of program registration documents: • Privacy of Personal Information Act (POPIA) to facilitate sharing of client data to facilitate linkage to additional preventative or treatment services • DREAMS informed consent • Sociodemographic data	HIV vulnerability screening assessment, pre and post-test counseling, HIV rapid test (HIV negative test result indicated for PrEP initiation), information sharing on PrEP, effective PrEP use and risk reduction counseling.	Pregnancy test (negative result inclusion criteria and indicated for PrEP ring as per national guidelines), STI screening, contraceptive and condom provision, PrEP method eligibility assessment, choice counseling incl. ring insertion and removal demonstration, demonstration tools/job aids (ring and pelvic model), creatinine clearance (eGFR) where appropriate, hepatitis B testing where appropriate, address side-effects, discuss PrEP continuation or discontinuation 3-month drug prescription, effective PrEP use and risk reduction counseling.	HTS, STI, and pregnancy screening; Cr clearance (eGFR) where appropriate; address side-effects; discuss PrEP continuation or discontinuation; 3-month drug prescription	PrEP drug (ring or oral PrEP)	1. HTS 2. STI and pregnancy screening 3. Cr clearance (eGFR) where appropriate 4. Address side effects 5. Discuss PrEP continuation or discontinuation 6. 3-month drug prescription

### 2.3 Measurement

To describe the implementation approach, data were extracted from various sources. Enrollment data from the routine patient management system (REDCap) were collected at implementation service points and data were collected from four focus group discussions conducted with implementation staff. The demographic variables included age, nationality, marital status, current schooling status, highest level of education completed, employment status, and site description. The behavioral variables included the number of current sexual partners, transactional sex, sex under the influence of alcohol and drugs, ever used PrEP, and the PrEP method chosen at enrollment. The process measures, collected through the routine service delivery tools, included implementation mode (mobile or gazebo), estimated time to deliver services including delivery of PrEP choice and counseling, and method of PrEP ring insertion (self, nurse, or assisted).

### 2.4 Analysis

STATA statistical software version 18.0 (Stata Corp, 2017; College Station, USA) was used for all quantitative analyses. Participant and implementation characteristics at enrollment were tabulated using frequencies and proportions. The sub-group analyses included PrEP method uptake, implementation modality, and ring insertion. Qualitative analysis was inductive, that is, observations were made, patterns recognized, and emergent themes formulated. Transcribed focus group discussion data were coded on NVivo v.14 and a thematic analysis was performed.

### 2.5 Ethics statement

The research was approved by the University of Witwatersrand Human Research Ethics Committee (Medical) (Reference # 230412). All the participants provided written informed consent. Participants were only reimbursed for study-related procedures, specifically participation in the baseline and follow-up surveys and focus group discussions. Compensation was not provided for routine clinic visits to avoid being an incentive thereby influencing clinic attendance and PrEP method continuation.

## 3 Results

### 3.1 Participant sociodemographic and behavioral characteristics

A total of 752 participants were screened. Of these, 659 (87.6%) were eligible, and 657 (99.7%) participants were enrolled in the cohort study between 03 October 2023 and 12 April 2024 across 38 community-based study sites. The majority (72.1%) of the participants were between the ages of 18 and 24 years, had South African citizenship (95.9%), reported single relationship status, and had completed secondary school (grade 12) education (80.2%). The majority (63.3%) were studying toward a tertiary education degree/diploma (63.3%). Furthermore, 75% of participants at enrollment reported never having used oral PrEP before. Regarding sexual and behavioral characteristics within the last 3 months, 2.1% of participants reported having transactional sex, 22.8% reported sex under the influence of alcohol, and 71.5% reported condomless sex. A third of participants (33.8%) reported a prior pregnancy and 44.6% reported no contraceptive method use at enrollment. Table 2 describes additional participant characteristics at enrollment.

**Table 2:** Participant characteristics at study enrollment in Johannesburg from 03 October 24 to 12 April 2024, *N* = 657.

Variable	Frequency (*N*)	Percentage
Age in years		
18–24	474	72.1
25–34	156	23.7
35 or more	27	4.1
Nationality		
South Africa	630	95.9
Democratic Republic of Congo (DRC)	1	0.2
Lesotho	3	0.5
Malawi	1	0.2
Mozambique	2	0.3
Zimbabwe	18	2.7
Other	2	0.3
Marital status		
Single	650	98.9
Divorced	3	0.5
Married	4	0.6
Current schooling status		
Never schooled	1	0.2
Out of school	222	33.8
Secondary	18	2.7
Tertiary	416	63.3
Highest level of education completed		
Primary (grade 7)	99	15.1
Secondary (grade 12)	527	80.2
Tertiary (diploma, certificate, or degree)	28	4.3
Missing	3	0.5
Employment status		
Employed	54	8.2
Part-time	9	1.4
Self-employed	13	2.0
Unemployed	580	88.3
Missing	1	0.2
Implementation site description		
College	100	15.2
Community^a^	315	47.9
Student residence/housing	30	4.6
Second chance matric centers	19	2.9
Technical, vocational, and educational training	159	24.2
University	34	5.2
Number of current sexual partners		
No sexual partner	80	12.2
One sexual partner	543	82.6
More than 1 sexual partner	34	5.2
Reported transactional sex in last 3 months		
Yes	14	2.1
No	633	96.3
Missing	10	1.5
Sex under the influence of alcohol in the last 3 months		
Yes	150	22.8
No	507	77.2
Sex under the influence of drugs in the last 3 months		
Yes	9	1.4
No	648	98.6
Condomless sex		
Yes	470	71.5
No	187	28.5
Ever had a sexually transmitted infection		
Yes	27	4.1
No	630	95.9
Reported ever intimate partner violence/gender-based violence experience		
Yes	6	0.9
No	651	99.1
Type of contraceptive method in use		
Intrauterine device (IUD)	11	1.7
Implant	68	10.4
Injection	135	20.5
Long-acting reversible contraception (LARC)	7	1.1
None	293	44.6
Pill	58	8.8
Missing	85	12.9
Ever used oral PrEP		
PrEP-naive	498	75.8
Prior PrEP user	159	24.2
Ever pregnant		
Yes	222	33.8
No	281	42.7
Missing	154	23.4

Percentages may not total to 100% due to rounding.

^a^ Community sites categorized as safe spaces operated by community-based organizations and youth skills centers.

The majority (68.7%) of the participants chose oral PrEP at enrollment, with just over a quarter (26.6%) selecting the PrEP ring. More than half (55.6%) of the participants received their PrEP method in the mobile truck, which was a slightly higher proportion compared to those who received PrEP in a gazebo (44.4%). The distribution of PrEP uptake and implementation modality is provided in Table 3.

**Table 3:** PrEP method uptake and ring insertion by implementation modality in Johannesburg at study enrollment, 03 October to 12 April 2024, *N* = 657

Variable	Implementation mode	Total	
	Gazebo, *N* = 291, 44.3%	Mobile van, *N* = 366, 55.7%	*N* = 657
	*N* (%)	*N* (%)	*N* (%)
PrEP method uptake			
PrEP ring	80 (27.5)	95 (26.0)	175 (26.6)
Oral PrEP	196 (67.4)	255 (69.7)	451 (68.6)
No method	8 (2.7)	12 (3.3)	20 (3.0)
Did not see a nurse^a^	7 (2.4)	4 (1.1)	11 (1.7)
Who inserted the ring^b^			
Nurse inserted	46 (57.5)	57 (60.0)	103 (58.9)^c^
Self-inserted at home by participant	31 (38.8)	32 (33.7)	63 (36.0)
Nurse assisted self-insertion at the site	3 (3.8)	6 (6.3)	9 (5.1)^d^

^a^ Participant left before seeing a nurse.

^^b^^ Among those who chose the ring.

^c^ Of these, 100/103 (97%) were inserted by a female provider.

^d^ Of these, 3/9 (33%) were inserted by a female provider.

Table 4 indicates that the majority of PrEP ring insertions (58.9%) were done on-site by a nurse compared to those who chose to self-insert at home (36.0%) or self-insert on-site with assistance from a provider (5.1%).

**Table 4:** Qualitative findings from FGDs conducted with providers by theme and sub-theme.

Theme	Subtheme	Quote
Preparedness to deliver PrEP	Training	“I think inserting the ring to the clinic… That was the most important part of the training because that is what is supposed to be done on site. You have to insert that ring. So, the demonstration of using that demo ring and the pelvic model of the vagina; the pelvic model where you insert the ring. Like showing us how it is done and the videos. That was the most important. (Professional nurse, female, 28 years) “I think the training was good. We were offered enough information for us to go out there in the community and be able to create or generate demand.” (Demand-creation mobilizer, male, 26 years) “We had the likes (of) doctor XXXX (name concealed). People who are qualified. We had the principal investigator. We had everyone there [at the training]. So we had access to every information we had and I feel like every question that we asked; they were answered on the sessions. And the fact that after the training we went to site and we came back to the training for feedback. We went back to site and then we came back for feedback doing continuous trainings. I think that helped a lot. (Demand-creation mobilizer, female, 31 years)
Setup and provision of PrEP through a community model	Service provision (acceptability/access to care) and reach (older vs. younger population)	“I think giving out PrEP in the community, it's more effective and efficient for most clients because they can just come in quickly and get all the services that they need; as to when to the clinic because they feel like they need to meet. It takes too long and the treatment at the same time is not okay.” (Lay counselor, male, 40 years) “In the community, it's more of like the older community that will reach out to you and be like, no, come, let's do this and do this. But obviously as our project, we have to prioritize the younger ones because that's what we are here for.” (Data capturer, female, 27 years)
	Challenges with implementation including continuation	“It is a good thing that we go to the community to assist those teenagers and our young women. But the point is after delivering, that is a good service; but then; some of them we are introducing them to those products like family planning injections and stuff. And we give them follow-up dates, but then we don’t go back to service them again. So it is like we are introducing them to something that we do not keeping up. We are not continuing with what we started with them. Some of them will only take that one shot and they do not go back to the clinic or they do not get any other shot for family planning; the contraceptive. So it is like we are introducing them to something that we are not following up and they end up defaulting to something that we introduced them to.” (Professional nurse, female, 28) “We can’t always go back on the same date because our spots are too many. There are those who are interested [in initiating PrEP]. Like, they would say I came but you guys were not there. So we restart, restart; restart [clients on Oral PrEP]. So we don’t know whether it is our fault or it is their fault. So if we see someone coming back for and we are not there.” (Professional nurse, female, 36 years)
	Provision of choice counseling and messaging (efficacy, ring insertion; drawing of blood; health talks)	“I, for one, I think, yes, I recommend the capsule rather than the ring. Well, the capsule, with the side effects maybe it's bad and it may make a client uncomfortable; whereas the ring; it only protects the lower part of the body and not the other. So what about, like I tell them about incidents where we contracting HIV without having sexual intercourse. So what's happening to those incidents because it comes back to me to say, “yes, I heard you say this, and then let's go back to the incidents you gave me with the ring, what must I do”?. You know? (Lay counselor, female, 34 years) “For me, it was a little bit difficult to actually not advocate or give awareness about oral [PrEP] only. I had to merge the two because at first, because it was new to me and we were still trying to see how we can come around it; so advocating the ring; now how the…the efficacy of it. So now I have to explain. It is like thirty-five percent, “oh, thirty five! It is not like I am not protected”! “No, you are because it can escalate to fifty percent; especially if you are not doing oral sex; anal sex.” But some would say “No, I am very adventurous when coming to sex.” So it becomes a little bit hard to actually; for participants to agree to take the other method. The dapi ring method.”(Demand-creation mobilizer, male, 32 years) “And another thing about the dapivirine ring is that, like my experience on the other side is that when you do health talks and you show the dapi ring, they are interested. But the problem is about the dapi ring, is that it is so…it is a silicone substance. When I make the demo, demonstration of dapi ring; it is a bit hard. For them it’s, they are skeptical. Especially about the size of the ring. Others would say can’t we make it a little bit smaller. I would say the cervix is wider, that is why the ring is a bit bigger.” (Demand-creation mobilizer, female, 35 years) “So, they [clients] feel like, even when you are giving out the message and you are explaining what the study is; what the study entails; they still have that; they convince themselves that we are trying to test the product on them. When you are trying to tell them that we have a ring. A ring, you do it under a study. The fact that you are mentioning a study is like you are testing the product on them. That is why most of them will not even take the method because they feel like now they are testing the method. Even when you try to explain that, “no, this method has been tested. It is working. Now we just want to see, gather the data about which method people prefer.” But they feel like, “no, we have never had anyone using this method. This is new and you are trying it out on us.” So, the first thing is that the community doesn’t understand when you try to explain what we are doing.”(Professional nurse, female, 28 years) “Mostly it {the fear of drawing bloods] happens with the clients that are taking oral PrEP. An example would be that they will get information about oral PrEP and all that, but when they come to me as a professional nurse; I would have to initiate. With oral PrEP, you need to draw blood for hepatitis B. So, that part; most of the client would not be comfortable drawing blood. So they will be interested that I want to take PrEP but the part of drawing blood; no. the other will give you reasons that probably previously; they drew blood and they collapsed. One, two; three happened so now they are not comfortable. Or they are just not comfortable. They are afraid of needles and I can’t force them. So I just need to explain to them that even though you are interested but I cannot draw blood; then I am not allowed to give you [oral] PrEP.”
	Time taken to offer and engage with the client regarding PrEP	“When you are explaining to them, they will not even focus. You can just tell them something now “okay, I said this.” You are trying to educate them. After some two seconds, you ask him or her “what did I say to you”? Doesn’t know anything. They will just say, “no sister; I am in a hurry” and all those things. “No, I have been here for so long” and all those things. “Your procedure is too long” and all those things. So…jah! And there is nothing we can do with the study. The procedure is a procedure” (Demand-creation mobilizer, male, 32 years)
Operational requirements for delivering community-based services	Resource availability (beds, toilets, water)	“In mobile vans you can see that some of the clients are not comfortable to be inserted the ring on the gazebo model. But it is better in the mobile because there is a bed and there is everything and also confidentiality [privacy] is maintained. So if, and also the advantage of the mobile is that they also have toilets because for vaginal ring, for a client to be eligible to be inserted the vaginal ring; that client needs to test negative for pregnancy. So for that, we need a toilet for clients to be tested. So, if you are using a gazebo model, there are no toilets. So you are depending on the community toilets…sometimes these toilets are far. So we need to go far with these clients for them to pee and test [for] pregnancy. But for mobile vans, mobile vans do have toilets and they have generators so they have everything. If everything is operating well in the mobile, then everything went well.” (Professional nurse, female, 36 years) “Also, in terms of resources as well, if we know this is going to be a community-based model, let this just be something we implement using trucks that have functioning toilets; finished. If you want it to be a community-based program, if you know that you gonna need to take a pregnancy tests, cars that must implement with teams need to be cars that have toilets” (Demand and linkage officer, female, 25 years).
	Delivery mode (mobile van vs. gazebo)	“Eh the problem is all the resources that we are using. I don't think… they are not suitable. I think sometimes our resources are the ones that discourage the clients to take PrEP, especially in a Gazebo, I won't feel comfortable [laughs] taking off my clothes in a Gazebo.” (Data capturer, 29 years) “So if it is possible, every nurse can have a mobile. That could also help even on the uptake of the ring. If we cannot partner with the facility, try to get more mobiles so that we know as a nurse, you have a mobile and the hygiene. Like, what our protocol needs and guides us. Like, you are not comfortable. You feel like you are doing something wrong if you are in a gazebo.” (Professional nurse, female, 36 years)
	System for making an appointment	“I don’t know if it’s possible but, I don’t know if it can work; but to work with appointments. We go to sites as demand and fieldworkers to recruit participants. And those that are interested, we book them. We know that today, we are just doing recruitment. For today and then we book another day, maybe on Wednesday or Friday we know that today we are only implementing. They are only getting services.” (Demand-creation mobilizer, male, 32 years) “I think for the ring, it would be appropriate for me if ever the clients were booking in advance; so we know how many clients we are going to see for that day because we are dealing with the most impatient people and for the ring, it has its own; I think it is the time.” (Professional nurse, female, 33 years)
Community engagement	Demand-creation tactics	“I want to mention social media. Because it's, I think it's one of our biggest gaps when it comes to getting engaged because once someone can identify you, they know exactly what's happening and they are interested, they know if this is for them or not. So social media, because social media right now, like in this day and age, it's very…like it holds that power in our day and age. If someone can see you on TikTok and they know that this is it, they're never like, okay, now I'm interested, like I understand what this is.” (Data capturer, female, 35 years) “Just to add on to what my colleague said, we do have radio slots. We do share the information and educate through radio. It is just that the message is not really forwarded to the right people that we are dealing with in our programme. But they do have slots in radio…But the right people or the right group that we want to spread the awareness to, they are on social media. They are on Tick Tock. They are on YouTube. That is where they spend most of their time at.” (Demand-creation officer, female, 25 years)

In some instances, there was a nurse provider preference to insert the ring at the enrollment visit to ensure that the method was *in situ* when the participant left and there ideally would not be any complications from having done it by themselves. At the follow-up visit, the participant would be capacitated to do self-insertion (illustrated by the below quote):

“They (the nurse provider) told me they must insert it, then they will teach me when I do the follow up. Then that’s when I will insert it by myself, that’s when I choose…. so that they can note whether I’m reacting fine or reacting or reacting bad to it.”

At the follow-up visit, the participant recalled her experience of inserting the ring on her own once the provider had instructed her on how to do it:

“Eish I was so nervous I couldn’t even insert it on my own [giggling] but she helped me then I managed to, now I insert it on my own in my own comfort at home.” (*Ring Participant, 19 years old)*

In contrast to the nurse’s preference for inserting the ring, some participants expressed the desire for the nurse to rather insert the ring at initiation than do it themselves for fear of not doing it correctly, as described below:

“…no, I asked the nurse that can she please insert it on me because myself, you will find that I am not doing the proper thing jumping around while I am inserting and then they told me that even though you are not comfortable even in your next appointment we going to do it again……like even when she inserted it, I am not feeling anything like I only heard her saying she has done.” *(Ring participant, 21 years old)*

On average, it took 22 min to conduct choice counseling, which was 7 min more than the counseling conducted in the context of routine service delivery where only oral PrEP is available.

### 3.2 Provider experience of and attitudes toward delivering community-based oral PrEP and PrEP ring services

Qualitative data were collected from 26 providers (12 clinical and 14 non-clinical) responsible for the delivery of PrEP choice. The team comprised 10 men and 16 women between the ages of 24 and 46 years with half having completed a tertiary qualification. Of the providers, 58% reported having more than 10 years of experience working in the HIV field. Focus group discussions conducted with the providers highlighted three broad themes and several sub-themes describing key implementation considerations for delivering the PrEP ring to participants through a community-based outreach model utilizing mobile trucks and gazebos. The first theme was *preparedness to deliver PrEP*, and under this theme, the providers shared positive experiences about the training and support received to introduce a new PrEP method and offer PrEP choice. Experience and perceptions of the *provision of PrEP through community outreach* were explored, and here, providers stated the value of using this model to reach young people, specifically AGYW who reported a fear of visiting, or inability to access, healthcare clinics for SRH services due to staff unfriendliness. Providers stated they were comfortable providing PrEP choice counseling, however, they noted that community-based counseling to AGYW posed challenges, such as impatience with the length of the process and poor continuation. Furthermore, the provision of an indicated PrEP method was influenced by participants' fear of needles and requirements for blood samples (as was the case with oral PrEP). Several *implementation considerations* were shared by providers, including considerations regarding messaging and demand generation, community engagement, and resource availability (access to running water and toilet facilities to conduct product screening and eligibility procedures).

## 4 Discussion

Our study demonstrates that delivering PrEP choice through a community-based model is feasible, noting that there are specific implementation requirements that need to be considered to ensure scalability, specifically the implementation modality (mobile vs. gazebo), approach to healthcare provider training, client preference for ring insertion (self, nurse, or assisted), community engagement and demand creation, and the resources required to ensure efficient and acceptable service delivery.

Nyblade et al. highlight that although there have been global efforts to scale youth-friendly services, barriers to accessing care for AGYW remain. Described as clinic stigma, AGYW are treated differently to other clients, made to wait longer for services, and are refused PrEP services to discourage sexual activity (20). Therefore, our model of decentralizing services from facility-based models of HIV prevention to non-stigmatizing and integrated services in community settings has the potential to increase access and meet the needs of clients where they are (21). A dapivirine ring situation and delivery channel analysis conducted in 2021 (22) found that, from a range of service delivery channels, the first priority for delivery of PrEP rings in South Africa is through public sector clinics and population-focused programs that have the best reach for women and girls at substantial risk for HIV and a high capacity to effectively deliver PrEP rings. However, the situational analysis noted significant variation in delivery models within public sector channels and emphasized the need for HIV prevention integration with family planning services, community-based/outreach services, and adolescent-friendly services that are particularly relevant for PrEP rings. The WHO also recommends that PrEP rings be delivered alongside oral PrEP, which would require relatively few additional components and it would, therefore, be feasible and appropriate to deliver PrEP rings in community settings (8).

Our study shows that providing PrEP rings in community settings is feasible, with the majority of the participants receiving the product in a mobile clinic consulting room. However, it also demonstrates that, when resources are constrained, interventions to increase access must be cost-effective. The delivery of PrEP choice can be provided with fewer resources (gazebos) without having to rely on high-value assets (trucks). This provides more flexibility to set up in difficult spaces without the need to navigate difficult terrain. However, the privacy and setup of service delivery spaces are important implementation considerations, in particular, the direction of facing, proximity to other clients who could overhear or have sight of procedures, and the cleanliness of the space, especially where vaginal exams/product insertions are taking place. The mobile clinic offers a more clinically suitable space for nurses to function, whilst the gazebo was perceived as less formal or inappropriate for clients to disrobe.

Fixed sites offer predictability to clients. Our study highlighted that a large number of sites, although effective for increasing reach and uptake, was not effective in ensuring continuation. Changing return dates and inconsistent access to community sites made it difficult to maintain a roster, thus affecting the continuation of methods. Our recommendation would be to focus on fewer high-volume AGYW sites to allow for better coordination and predictability for clients.

Our study did highlight provider preference, particularly with regard to messaging on the efficacy and location of action of the products. Therefore, healthcare provider training, particularly supporting the introduction of new PrEP products, requires an upfront investment of time and face-to-face training, with ongoing experiential mentoring and supervision.

The provision of choice counseling is complex and requires approaches to engage young women to support their informed choices. Specifically, approaches to keeping them engaged during counseling sessions are required, thereby allowing them to understand their prevention needs, and choose a PrEP method most suitable to their concerns, lifestyle preferences, and ability to effectively use and continue. Moreover, our study showed that the participants who reported lesser behavioral vulnerability (no sexual partner) to acquiring HIV still opted to initiate PrEP. This could be explained by findings from Celum et al. (23) who reported that participants may join studies to gain perceived better quality of care and faster services, in comparison to crowded public healthcare facilities. Furthermore, Rousseau et al. (24) also reported that AGYW with perceived HIV vulnerability due to fear of experiencing sexual violence in their communities were more likely to take up PrEP.

Our study showed that young women preferred for providers to insert the PrEP ring at enrollment despite the availability of a demo ring and pelvic model to illustrate the process of insertion and removal along with client-facing materials, which included a QR code linking the participant to images, and a video demonstrating the process. Acknowledging that this paper reflects participant preference at enrollment, it is important to note that Montgomery et al. (25) demonstrated that with gradual participation in the trial, participants reported ease of use and integration into their lifestyles. Therefore, the acceptability of the ring increased as participants became more familiar with the product. However, at this initial visit, the participants expressed an initial uncertainty about inserting the ring for fear of not doing it correctly and therefore relied on the nurse to insert the ring at initiation with the opportunity to be further capacitated at follow-up visits. We also noted that despite having a demo ring and pelvic model for illustration purposes, the size and rigid silicone of the ring (which seemed less pliable than the actual product) was also a concern for participants and this was a deterrent to self-insertion or uptake of the method. Experience with similar products is an important consideration driving choice. In this predominantly young group of women, with the majority between the ages of 18 and 24 years, it is important to note that experience with an intravaginal product was low, as was seen in the contraceptive field where the majority preferred to use an injectable contraceptive method compared to other methods (26).

As countries think through approaches to de-medicalize PrEP delivery, a move toward client-centered and self-care options, supporting user autonomy and confidence to insert or inject, as may be the case with sub-cutaneous injectable PrEP (lenacapavir), will be important.

Client education and messaging about the efficacy and characteristics of the product are important. Our data highlighted that the size of the PrEP ring, the silicone substance, efficacy, and site of action (systemic or localized) were potential concerns reported by clients to providers. Providers therefore need to be adaptable and knowledgeable to facilitate informed choice counseling. This is particularly noteworthy for staff who are tasked with demand creation (peer navigators, peer educators, demand-creation officers, and mobilizers), who are often the first people to come into contact with potential end users and may influence method choice solely through the type of information shared. Although every effort was made to standardize messaging and use national job aids and demand-creation materials, translation to implementation may not have always been consistent. Therefore, in addition to the standardized implementation materials and job aids, we also recommend more experiential training and ongoing mentoring to ensure consistency of messaging and practice responding to frequently asked questions encountered in the field to ensure that information is provided in a non-biased way. Furthermore, informed choice counseling should not only be centered around PrEP but also the integration of PrEP with SRH services. Our data underscores the importance of integrating PrEP with broader SRH services, particularly with the high number of those who reported condomless sex. Through this approach, clients have the opportunity to align their prevention journeys for better overall wellness outcomes.

The product eligibility requirements of the PrEP ring in South Africa made it difficult to implement it in community settings where there was no or limited access to water. However, this may be less relevant in other countries where the PrEP ring is indicated for use in pregnant (Lesotho and Kenya) and breastfeeding (Zimbabwe) women. With the recent release of data regarding the safety of the PrEP ring in pregnant and breastfeeding populations (27), updating the country guidelines to remove the requirements for pregnancy screening could remove barriers to implementation and make delivery in community-based settings more feasible.

## 5 Limitations

We observed missing data for some participants (*n* = 11) with respect to method choice and implementation modality. We postulate that the clients with missing method choice information were issued with oral PrEP or no method as the number of PrEP rings was accounted for on a study-specific accountability log. These clients were not recorded in the accountability log. However, we could not be certain of the method provided and therefore these were included as missing. Concerning missing information for implementation modality (gazebo or mobile truck), it is likely that the professional nurse, responsible for the completion of this data field, did not complete this field. Given that this study was conducted as a real-world service delivery program leveraging routine data systems and multiple reporting tools, it is unsurprising, though limiting, to have some level of missing data. We also would like to note that whilst every effort was made to return to sites within the follow-up window, the predictability of return was not always consistent. This was mainly due to the difficulties with implementing a rigid scheduling roster for 36 sites, flexible operational hours of community-based sites, the amount of resources (providers and implementation teams) available on a given day, and balancing requests between study sites and existing DREAMS program sites. As such, we acknowledge that this may have affected the continuation rates reported in the study.

## Data Availability

The original contributions presented in the study are included in the article/Supplementary Material, further inquiries can be directed to the corresponding author.
